# Pneumonia a neglected disease: A mixed-method study on the case-finding program in Indonesia

**DOI:** 10.3934/publichealth.2020008

**Published:** 2020-02-06

**Authors:** Sulistyawati Sulistyawati, Liena Sofiana, Sholehatun Khairul Amala, Rokhmayanti Rokhmayanti, Fardhiasih Dwi Astuti, Desi Nurfita

**Affiliations:** Department of Public Health, Faculty of Public Health, University of Ahmad Dahlan, Yogyakarta, Indonesia

**Keywords:** program evaluation, Pneumonia, case finding

## Abstract

Pneumonia eradication program has been implemented, but the incidence rate remains high. This research aims to evaluate the Pneumonia finding program in Sleman district of Indonesia. A mixed-method using sequential explanatory design was conducted during July–August 2019. Input, Process and Output were used as the evaluated methods. Input component were human resource quality, facilities, infrastructure and funding. Process aspect was planning, implementation, monitoring and evaluation. Output was the Pneumonia finding coverage. The quantitative study that employed a survey was done among the Pneumonia officer in 25 primary health centres in the research area. Qualitative study used a semi-structured interview to explore the Pneumonia officer's opinion about the case finding program. Analysis was performed in two stages: 1) Quantitative analysis was started with input data to the spreadsheet, clean the data, and classify into the cut-off. 2) Qualitative analysis was done using a content analysis approach. Input and process found sufficient. But we found poor in the output (Pneumonia finding coverage). Pneumonia finding program has not executed as the expected. The coverage was inadequate since only one out 25 reaches the target of Pneumonia finding coverage. Pneumonia is considered not severe disease become a reason for the inadequate coverage.

## 1. Introduction

Pneumonia caused by viruses, fungi or bacteria [1], becomes a significant killer for children diseases globally [2]. This disease received global and severe attention from many countries in the globe due to the large children infected. UNICEF stated that every year, more than 1400 cases per 100,000 children reported suffered from Pneumonia which most of them was reported happened in South Asia (Incidence rate 2500 cases/100,000 children) also West and Central Africa (1620 cases/100,000 children) [3]. In addition, World Health Organizations stated, Pneumonia was responsible for 808,694 children mortality in 2017. Fifteen per cent of all mortality was occurred among children under five years [1].

Pneumonia mostly occurs in a developing country with a fragile health system because of the complexity [4]. Pneumonia is preventable through immunization, food nutrition, and modifying the environment [1]. However, these approaches may challenge for a country with an unstable health system. Recognize the Pneumonia symptoms earlier are the effective approach to prevent the fatality among the children.

Pneumonia is one of Acute Respiratory Infection (ARI) which a severe problem in a developing country including in Indonesia. Every year, ARI is the top ten disease leading to death, especially in children under five [5]. In 2014, the center for research and development of Indonesia reported 9.40% mortality among children under five. In 2015, the case finding for Pneumonia was 63.45% [6]. Indonesia Ministry of Health stated in 2019, the number of Pneumonia incidence has increased [7]. To respond to this situation and prevent the mortality the Indonesia government has encouraged integrated management of childhood illness (IMCI). IMCI conducted passively in PHC aimed to perform early diagnosis for children under five, improve management, health promotion and increases mother knowledge. This program covers several diseases such as Pneumonia, diarrheal, measles, malaria, ear infections and malnutrition.

Integrated management of childhood illness was introduced in 1996 adopted from WHO and UNICEF program to reduce mortality in children under five [7],[8]. This program screens the children with medical check-up by skilled health care attendance. Integrated management of childhood illness must be conducted in a primary health centre. However, the implementation encountered some problems. This condition affects the coverage of Pneumonia case finding that has been set up by the government.

In 2018, the national target for Pneumonia case finding was 80%, meaning that each province has to achieve that target. However, until the report had been released, only Jakarta had achieved the national target (1 out of 34 provinces) [9]. Various situation and condition among the region in Indonesia, such as the different geographical condition, every district has the autonomy to set up their target based on their real situation. Yogyakarta province puts serious attention to the Pneumonia disease. This province consists of five districts, one of them is Sleman that struggle from Pneumonia in children. Their coverages for Pneumonia finding was 42.6% in 2018. This number was below the Yogyakarta province target (46.4%) and Sleman district target was 60% referred to Sleman officer. Based on these facts, assessing the Pneumonia case finding in children under five programs in the primary health center is essential to perform.

Input, process and output (IPO) can be used to evaluate the particular health program [10]. Input consists of every resource as the capital point to execute the program. The process is how the program implemented. The output is the core indicator and measurement to measure the health program labour [11]. This study aimed to assess the pneumonia finding program implementation in Sleman district of Yogyakarta.

## 2. Materials and method

### 2.1. Research design

A mixed-method using sequential explanatory design [12] was conducted in Sleman district of Yogyakarta during July–August 2019. In 2017, 1,062,861 people reside in Sleman district who administratively spread into 17 sub-districts with a total area is 57,482 km^2^ [13],[14]. Health services in Sleman are delivered by 25 primary health centre and more than ten hospitals.

**Figure 1.**
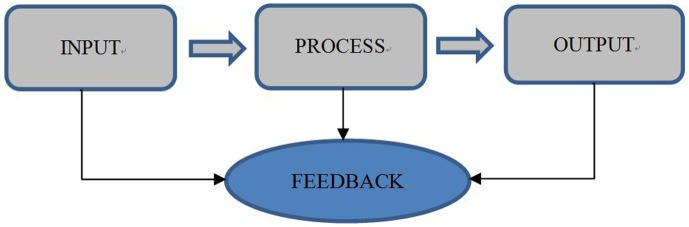
The framework of research.

This study investigated the implementation of Pneumonia case-finding program in children in Sleman district during January–December 2018. Input, Process and Output were used to evaluate the program (Figure 1). Input consists of human resource quality, facilities and infrastructure and funding. The process comprises of planning, implementation, monitoring and evaluation. The program output is case finding coverage.

### 2.2. Study participants

In the quantitative study, we applied a total sampling approach by recruiting the pneumonia officer in the entire primary health center in Sleman district. An invitation letter was sent to each PHCs to get permission from the head of PHC. After that, we asked about the willingness to participate in this study to the Pneumonia officer. When the agreement has been reached, the questionnaire was distributed for collecting the data. After the quantitative analysis was completed, the result was used to guide the selection PHC selection for the qualitative study. In-depth interview with Pneumonia officer was held in four PHCs that representing PHC with high and low Pneumonia finding coverage. To get balanced information, three officers from district health office who worked relate to Pneumonia were invited to join in this study.

A survey was conducted in 25 PHCs in Sleman to collect the information from the Pneumonia officer. A set of structured questionnaires was used to generate the data from the respondent. This instrument was developed according to the Indonesian national Pneumonia book program that used as a reference in PHC. Questionnaire was divided into three main sections: 1) identity and characteristic respondent, 2) input that asked about human resource quality, facility and infrastructure and funding, 3) process consist question about planning and implementation. Output as the component of the evaluation was collected using secondary data from each primary health center.

In a qualitative study, a semi-structured interview was directed to explore the finding in the quantitative phase. Oral and written informed consent, along with recording the interview was acquired before the in-depth interview. We asked the Pneumonia officer opinions about the course of pneumonia case finding in their PHC, does the case finding worked in a proper way, the challenge they faced on the pneumonia case finding. Probing to the informant was done when the questions need to be improved.

The research group conducted data collection. Prior to the fieldwork, a workshop was held among the people involved to train the field worker on the questionnaire and in-depth interview guideline.

### 2.3. Analysis

The analysis was conducted in two stages: 1) Quantitative analysis was analyzed descriptively started with data input to the spreadsheet, data cleaning, data calculation and visualization. 2) Qualitative data were analysed using a content analysis approach [15]. All interviews were verbatim transcribed in Bahasa Indonesia. Merging between Quantitative and Qualitative were done using building approach [12]. We selected the primary health center based on the level of Pneumonia finding coverage: high and low. The result among the two studies was interpreted together with compare and contrast technique.

### 2.4. Ethical consideration

This study was approved by the Ethical Review Board of Universitas Ahmad Dahlan, Yogyakarta, Indonesia (ethical approval code: 011906063). Research permission was obtained from the Sleman Health Office. Written consent was obtained from the respondent before they enrolled. Participants were informed, they have the right to quit from the study at any time and for any reason.

## 3. Results

### 3.1. Quantitative study

#### 3.1.1. Characteristic respondent

Twenty-five pneumonia officer had participated in the survey were mostly woman (92%). Majority of the respondents were had been working for more than two years in the associated public health centre (Table 1).

**Table 1.** Characteristic of the participants for the quantitative study in twenty-five public health centre in Sleman District.

Respondent		N (%)
Sex	Male	2 (8)
	Female	23 (92)
Main position	Nurse	22 (88)
	Midwife	1 (4)
	Epidemiologist	1 (4)
	Sanitarian	1 (4)
Work duration	≤ 2 years	11 (44)
	>2 years	14 (56)

#### 3.1.2. Evaluation of pneumonia case finding: input, process, output

**Figure 2.**
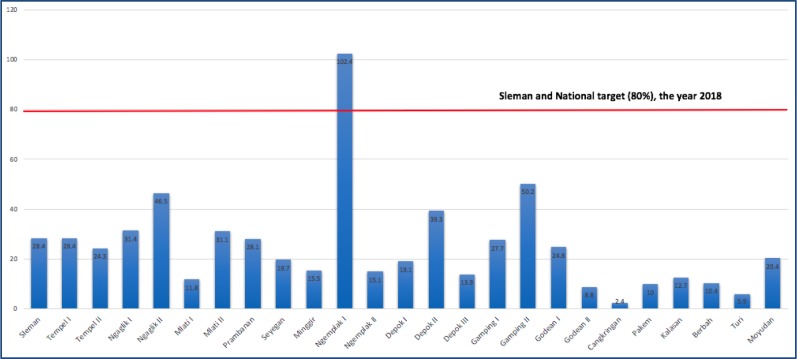
OUTPUT, comparation between pneumonia finding coverage by primary health centre versus Sleman district and national target year 2018. (Data Source: Sleman District Health Office, 2019).

Table 2 and 3 are presenting Input and Process of Pneumonia finding case program, respectively. Most of the components are have sufficient percentage. Output found insufficient, only 1 out of 25 PHC reached the target (4%) (Figure 2).

**Table 2.** INPUT components of pneumonia finding case program.

Human resouces quality	Yes. N (%)	No. N (%)
The presence of Pneumonia officer at the health centre	25 (100)	0
The involvement of Pneumonia officer in the pneumonia training	16 (64)	9 (36)
Pneumonia officer has double positions	19 (76)	6 (24)
Facilities and infrastructures	Yes. N (%)	No. N (%)
The primary health centre has oral and injection medication	25 (100)	0
Availability of ARI sound timer in the primary health center	16 (64)	9 (36)
The availability of nebulizer in the primary health center	24 (96)	1 (4)
The availability of pneumonia reporting forms for children under five	25 (100)	0
Availability of media promotion at the the primary health center	18 (72)	7 (28)
Funding	Yes. N (%)	No. N (%)
The sufficiency of funds	16 (64)	9 (36)
The funding source of Pneumonia programs	Government N (%)	Do not know N (%)
	15 (60)	10 (40)

**Table 3.** PROCESS components of pneumonia finding case program.

Planning	Yes (N%)	No (N%)
The existence of pneumonia program plan	16 (64)	9 (36)
Implementation	Yes (N%)	No (N%)
Implementation of Pneumonia health promotion	16 (64)	9 (36)
Implementation of Pneumonia care-seeking in children under five	6 (24)	19 (76)
IMCI implements routinely in children under five at primary health center	16 (64)	9 (36)
Cross-program collaboration in the primary health center	21 (84)	4 (16)
Cross-sector collaboration in the primary health center	11 (44)	14 (56)
Availability of information communication counselling education Pneumonia in children	25 (100)	0 (0)
Monitoring and evaluating	Yes (N%)	No (N%)
Monitoring and evaluation was realised	25 (100)	0 (0)

Figure 2 shows the comparations the case finding target between PHC, District and National year 2018. Among the 25 primary health centres in Sleman, only one PHC achieve the target set and the rest were below for both the district and national target.

### 3.2. Qualitative study

#### 3.2.1. Characteristic informants

Seven informants have participated in the qualitative study, representing the stakeholder involved in Pneumonia disease. Four of them work in the primary health center, and three informants work in the district health office (Table 4). We reached the saturation after accomplished the interviews.

**Table 4.** Characteristics respondents for the qualitative study.

Position	Education	Age	Informants code
Division Head of Disease Prevention and Control in Sleman District Health Office	Doctor	53	3
Section Head of Infectious Disease Prevention and Control in Sleman District Health Office	Nursing	56	2
Pneumonia officer in Sleman District Health Office	Lab. Analyst	46	1
Pneumonia officer in Ngaglik II PHC	Public Health	27	4
Pneumonia officer in Prambanan PHC	Nursing	40	5
Pneumonia officer in Pakem PHC	Sanitarian	26	6
Pneumonia officer in Cangkringan PHC	Nursing	50	7

#### 3.2.2. Challenge in pneumonia case finding

Seven in-depth interviews were completed, four represents the primary health center, and three interviews were done in the district health office. Our analysis produced six subcategories, three categories and one theme entitled Pneumonia as a neglected disease. This theme figures out pneumonia received lack of attention from the stakeholders involved due to management gap, knowledge gap and insufficient planning (Table 5). We are presenting the subcategory (in bold) and category followed by the example quote from the respondent.

**Table 5.** Illustration of subcategory, category and theme.

Subcategory	Category	Theme
Having double positions	Management gap	Pneumonia a neglected disease
Insufficient officer number		
Insufficient room for IMCI		
Insufficient knowledge due to inadequate training	Knowledge gap	
Program planning was not based on data	Insufficient planning	
Rising stigma – Pneumonia not a severe disease		

#### 3.2.3. Management gaps

This category consists of two subcategories “Pneumonia officer have double positions”, “insufficient officer number” and “insufficient room for IMCI”. The respondent said that they could not handle all disease that becomes their responsibility, and this situation makes them hard to focus on a specific disease, including Pneumonia that considers as not severe disease.

*“The person in charge of Pneumonia is one person. But, here I do not only handle Pneumonia but other infectious diseases also: diarrhoea, leptospirosis, dengue, TBC and HIV”*. (Pneumonia officer 5).

*“If I am only responsible for Pneumonia, maybe I will more focus…the important thing is not a double job. It is tough full for me – double burden”*. (Pneumonia officer 7).

*“Here, Pneumonia is arbitrary. I am a nurse, but I am also an IMCI program implementor”*. (Pneumonia officer 7).

Some respondents elaborated about the insufficient number of officers in their PHC. It is correlated with work loading because every day PHC receives an enormous patient.

*“The number of patients in PHC is huge; you can see in PHC only have 2 doctors, even some PHCs only have 1 that have to serve about 100 patients with several symptoms. Impossible to conduct a fast examination”*. (Pneumonia officer 2).

*“Indeed, for diagnosing Pneumonia relies on the human resource, we have trained the new Pneumonia officer, but the patient comes a lot, so we need more time for diagnosing the patient”*. (Pneumonia officer 3).

*“The challenge to find a Pneumonia case is the limitation of human resource. We suggest building a team”*. (Pneumonia officer 6).

Respondents express that IMCI could not be held properly because there was not sufficient room. On the other hand, in some circumstances, the patient requires specific treatment such nebulizer, which it needs comfortable space.

*“The obstacle to conduct IMCI was insufficient room though we had a lot of as a patient where we need to counting breaths. If we entered them to the general polyclinic, the situation would be crowded”*. (Pneumonia officer 5).

#### 3.2.4. Knowledge gaps

Some Pneumonia officer spoke about the “insufficient knowledge due to insufficient training”.

*“Training not yet…if socialization yes (once) on how to input the data”*. (Pneumonia officer 6).

*“I don't have a plan for Pneumonia because I don't know how to execute it”*. (Pneumonia officer 6).

#### 3.2.5. Insufficient planning

Insufficient planning arises from subcategory “data utilization for planning purposes is low” and “rising stigma-Pneumonia not a serious disease”. Pneumonia officer felt the utilisation of data they are collected is low to build the Pneumonia program.

*“We have a comprehensive data, but still challenging to use the material for planning purposes”.* (Pneumonia officer 6).

Pneumonia disease is considered not as a severe disease. Accordingly, Pneumonia is ignored.

*“Hard to put my time on Pneumonia because if we see, the mortality of pneumonia is low compared to, for example, leptospirosis”.* (Pneumonia officer 5).

*“Pneumonia receives low attention, ignores by the Pneumonia officer. This is our problem”.* (Pneumonia officer 1).

## 4. Discussion

It is recently reported as the most disease that causes death in children under five is Pneumonia. According to UNICEF, this disease responsible for the mortality of one child every 39 seconds [16]. Nowadays, Pneumonia program already set up by the Indonesia ministry of health and delivers from provincial health office to the primary health centre. However, the person in charge also handles for some other responsibility in the PHC. This mechanism triggers the Pneumonia program output was unclear. Exploring the mechanism of integrated management of childhood illness through Input, Process and Output (IPO) will facilitate the development of Pneumonia program in the future by improving the currently available program.

This research identified some weaknesses in pneumonia case finding implementation. It can be seen from the result that input and process were useful, but they had a poor output almost in the public health centre. But when we compared this result with the in-depth interview result, Pneumonia unluckily considers as a neglected disease in the research area. This situation, actually not only in Indonesia, as confirmed by the UN that Pneumonia as the killer disease in the world [17].

Management gap has a contribution to the problem. Having a multi-position due to the insufficient officer produced not optimal performance though mostly respondent said that the human resource was sufficient. Some respondents stated that the lack of person influenced their Pneumonia program. It was consistent the high demand for work could end up with exhaustion and finally will reduce the work quality [18],[19]. This situation cannot be ignored because it produces an unexpected impact on the program. Assessment of the work among the Pneumonia officer should be performed to assess the actual workload.

Proper knowledge is essential to support the health care system succeed [20]. This knowledge can be gained from formal education and training. Insufficient expertise due to lack of training led failed to recognize their responsibility about Pneumonia prevention and finding. This could be when discussing program sustainability. The rotation of people in the working area is a tough situation for people who positioned in different place of expertise when they move to a new place. When it does not support the proper environment, the rotation might give a negative effect. For example, when the turning is conducted in irregular time. The associated Pneumonia officer has no time to follow the training, ignoring the Pneumonia program.

Using data for developing a health program by the decision-maker is mandatory [21]. To develop a proper program requires time and accurate information [22]. Even though in the survey, most of the respondent discussed IMCI planning. However, a contrast result was found in the qualitative result. The data useless on the Pneumonia program influenced the quality of the planning inaccurate, inefficient and cost consuming. The second reason why Pneumonia has an insufficient plan is related to the stigma that Pneumonia is not a severe disease. This result consistent with the improper room for IMCI activity that affected both the health care attendance and patient. Then, we can question the commitment of the decision-maker on providing excellent health care services.

This study may have some limitations. First, in a quantitative study, self-report questionnaire allows the respondent to response with the normative (positive) answer, then the actual problem was not identified. Second, in a qualitative study where some informants were occupying a new position as Pneumonia officer. So, some of them could not address our answer completely. The future research proposed to conduct an in-depth assessment of the Pneumonia officer performance with the current situation to explore the potential improvement.

## 5. Conclusion

In summary, Pneumonia case-finding program in Sleman, Yogyakarta has not implemented as the expected. The coverage of the case finding was inadequate since only one out twenty-five PHC reached the target. Ignoring the Pneumonia impact and having an opinion that Pneumonia is not severe disease causes that Pneumonia case finding implement incorrectly.
